# Impact of serum uric acid to high-density lipoprotein cholesterol ratio on short-term outcomes in acute decompensated heart failure: a cohort study in Jiangxi Province, China

**DOI:** 10.3389/fendo.2025.1667929

**Published:** 2025-10-07

**Authors:** Zihao Lu, Guoan Jian, Kun Jiang, Shiming He, Zhenyu Wang, Juan Wang, Houhui Lan, Guotai Sheng, Yang Zou, Shuhua Zhang

**Affiliations:** ^1^ Jiangxi Medical College, Nanchang University, Nanchang, Jiangxi, China; ^2^ Jiangxi Cardiovascular Research Institute, Jiangxi Provincial People’s Hospital, The First Affiliated Hospital of Nanchang Medical College, Nanchang, Jiangxi, China

**Keywords:** uric acid to high-density lipoprotein cholesterol ratio, heart failure, acute decompensated heart failure, uric acid, high-density lipoprotein cholesterol

## Abstract

**Introduction:**

Accumulating evidence suggests that both serum uric acid (UA) and high-density lipoprotein cholesterol(HDL-C) play critical roles in the pathogenesis of heart failure. This study aimed to investigate the association between the UA-to-HDL-C ratio(UHR) and short-term all-cause and cardiovascular mortality in patients with acute decompensated heart failure(ADHF).

**Methods:**

A total of 2,404 ADHF patients admitted to Jiangxi Provincial People’s Hospital from 2018 to 2024 were included in this study. The association between the UHR and 30-day all-cause and cardiovascular-specific mortality in patients with ADHF was systematically evaluated using Kaplan-Meier analysis, Cox regression, restricted cubic spline models, and stratified analysis. The robustness of the findings was further validated through multi-faceted sensitivity analyses.

**Results:**

During the 30-day follow-up period, 156 patients(6.49%) died in the entire cohort, with 120 deaths attributed to cardiovascular causes. The all-cause mortality rates across UHR quartiles were as follows: Q1: 3.83%, Q2: 4.16%, Q3: 6.82%, Q4: 11.15%, while cardiovascular mortality rates were Q1: 2.33%, Q2: 3.49%, Q3: 5.32%, Q4: 8.82%. Multivariable Cox regression analysis revealed that each 1-unit increase in UHR was associated with a 30% increased risk of both all-cause and cardiovascular mortality in ADHF patients. Furthermore, compared with patients in the lowest UHR quartile, those in the highest quartile had an 88% increased risk of 30-day all-cause mortality and a 113% increased risk of cardiovascular mortality. Further restricted cubic spline regression analysis demonstrated a linear positive association between UHR and the 30-day risks of all-cause and cardiovascular mortality in ADHF patients. Stratified analysis revealed that the association between UHR and mortality in ADHF patients was not modified by age, gender, New York Heart Association classification, left ventricular ejection fraction, or comorbidities. Finally, multiple sensitivity analyses conducted across four dimensions—population heterogeneity, causal temporality, model adjustment, and data integrity—confirmed the robustness of the primary findings.

**Discussion:**

In this cohort study conducted in Jiangxi, China, we demonstrated for the first time that the UHR could serve as a tool for early prognostic assessment of short-term all-cause and cardiovascular mortality risk in ADHF patients, and elevated UHR levels were independently associated with an increased risk of both outcomes.

## Graphical Abstract

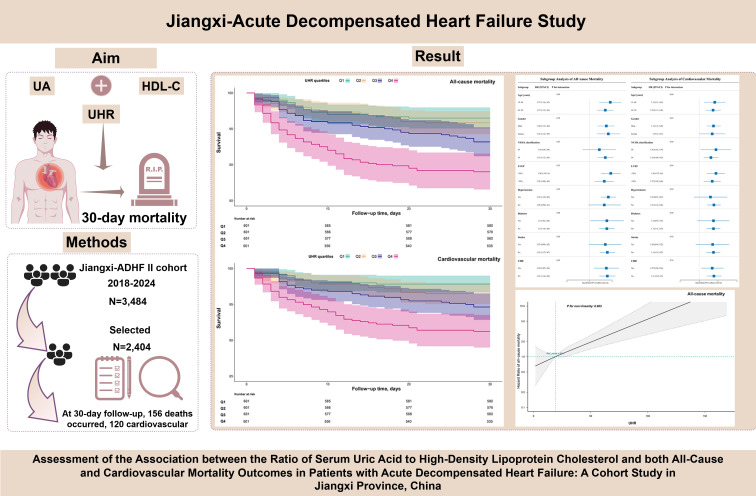

## Background

Acute decompensated heart failure (ADHF) is a cardiovascular syndrome characterized by sudden worsening of HF symptoms, primarily manifesting as circulatory congestion and organ hypoperfusion due to inadequate cardiac output, which can be life-threatening in severe cases (1–3). According to statistics, ADHF patients have a short-term mortality rate of approximately 12%, a 1-year readmission rate of about 45%, and a 1-year mortality rate of approximately 22% (4, 5). Despite ongoing advancements in treatment strategies, the incidence of short-term adverse outcomes in ADHF patients remains persistently high (6, 7). Therefore, given the significant short-term mortality and adverse prognostic features observed in ADHF patients, establishing an effective early warning system and promptly identifying critical risk factors driving disease progression may hold substantial clinical value for optimizing clinical decision-making and reducing short-term mortality.

UA (Uric Acid) and HDL-C (High-Density Lipoprotein Cholesterol) are two common metabolism-related indicators closely associated with cardiovascular health; research indicates that elevated UA levels significantly increase the risk of all-cause mortality in HF patients, and this association is consistent across both acute and chronic HF patients (8–14). HDL-C is a cardioprotective lipid. On one hand, it exhibits anti-atherosclerotic effects by promoting reverse cholesterol transport to the liver for metabolic clearance, thereby reducing atherosclerotic burden (15, 16). On the other hand, HDL-C possesses anti-inflammatory and antioxidant properties, protecting the cardiovascular system by mitigating oxidative stress and inflammatory responses (17–20). Clinical studies have demonstrated that reduced HDL-C levels are independently associated with an increased risk of HF (21–24). It should be noted that there is an interplay between UA and HDL-C, which is associated with future mortality risk in cardiovascular patients (25, 26). Recently, Kocak et al. proposed a ratio combining UA and HDL-C (UA to HDL-C Ratio, UHR) for the diagnosis of metabolic syndrome (27). Subsequent related studies have further revealed that UHR can also be applied to risk assessment for various cardiovascular diseases, including ischemic heart disease (28), hypertension (29, 30), and acute myocardial infarction (31). Notably, in a recent cross-sectional study, researchers found that elevated UHR is significantly associated with an increased risk of congestive HF (32). These findings collectively support a significant association between UHR and cardiovascular diseases (28–32); however, it remains unclear whether UHR can be applied to assess HF prognosis. Therefore, based on the above research background, this study aims to systematically evaluate the clinical association of UHR with 30-day all-cause mortality and cardiovascular-specific mortality in ADHF patients, using a cohort of ADHF patients from Jiangxi, China.

## Methods

### Study population and design

Data for this study were derived from the Jiangxi-ADHF II project. Briefly, the Jiangxi-ADHF II project is a retrospective cohort study that consecutively enrolled 3,484 ADHF patients admitted to Jiangxi Provincial People’s Hospital between January 2018 and January 2024. The project aims to investigate key factors influencing short-term adverse outcomes in ADHF patients, with the goal of refining early risk stratification strategies for this population. In this project, the definition of ADHF referred to the latest guidelines from the American College of Cardiology/American Heart Association and the European Society of Cardiology for HF (33–36), taking into account the subjects’ clinical symptoms, signs, and laboratory test results, primarily as follows: (1) At least one symptom worsened from baseline: (a) dyspnea (exertional dyspnea, paroxysmal nocturnal dyspnea, or orthopnea), (b) systemic venous congestion (lower extremity edema, hepatic congestion, or ascites), and (c) tissue hypoperfusion (oliguria or anuria, cold extremities, impaired consciousness, hyperlactatemia, or metabolic acidosis); (2) At least one sign of HF: (a) pulmonary edema detected by physical examination or chest X-ray, (b) elevated levels of B-type natriuretic peptide or N-terminal pro-B-type natriuretic peptide (NT-proBNP), and (c) echocardiographic evidence of cardiac structural and/or functional abnormalities.

**Figure 1:**
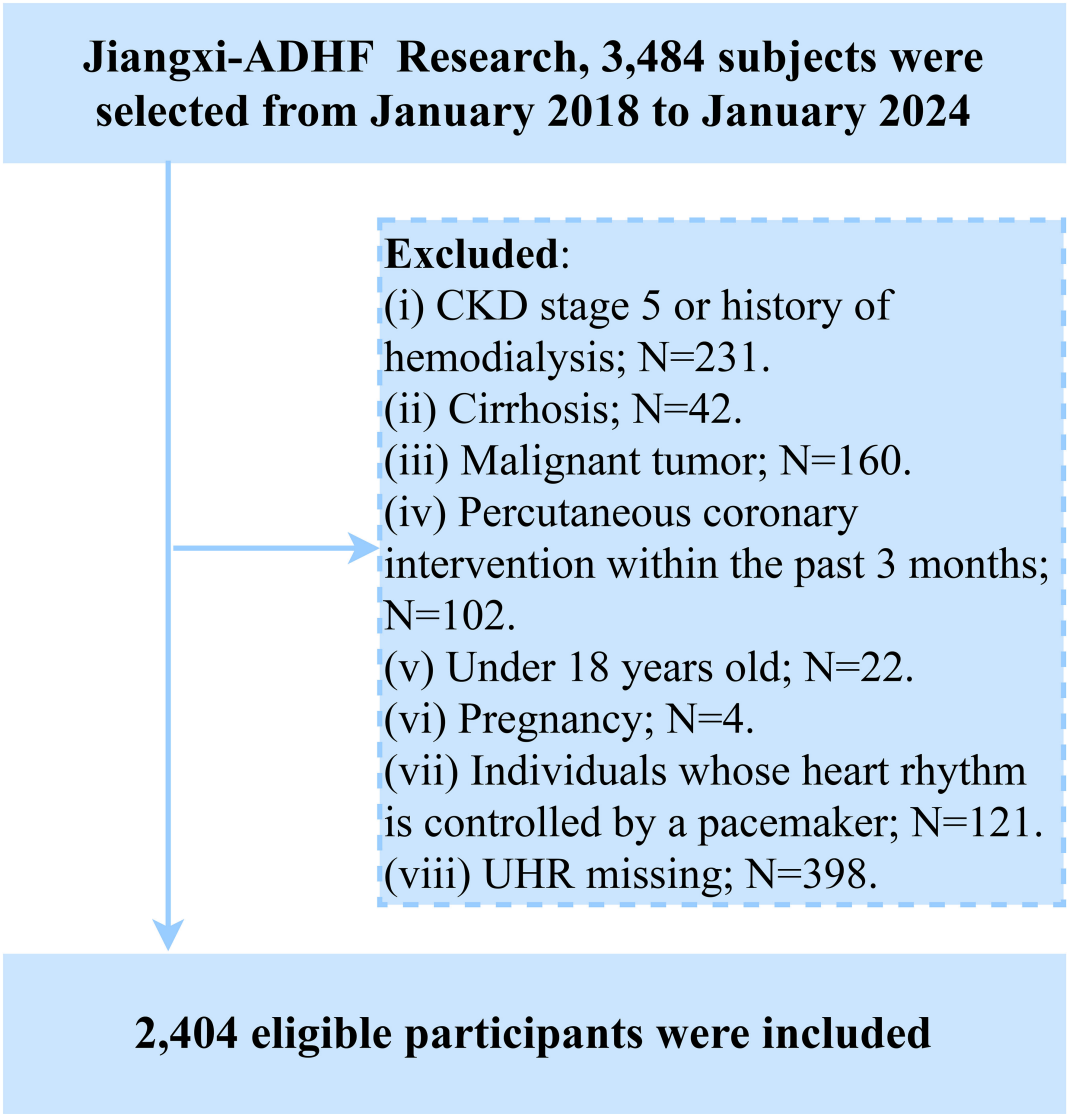
Flow chart for inclusion and exclusion of study participants.

Participants with the following baseline characteristics were excluded: (i) Those with malignant tumors (n=160) were excluded due to a potentially significant compromise in life expectancy; (ii) To mitigate confounding effects of fluid and sodium retention arising from other diseases, we excluded patients undergoing hemodialysis for chronic kidney disease or those with diagnosed uremia (n=231), and individuals with cirrhosis (n=42). (iii) Participants with pacemakers were excluded due to reliance on pacemaker-regulated heart rate (lack of intrinsic autonomic control) (n=121). (iv) To account for the significant impact of reperfusion therapy on short-term prognosis, individuals who underwent percutaneous coronary intervention within 3 months prior to admission were excluded (n=102). (v) Participants aged <18 years were excluded (n=22). (vi) Pregnant participants were excluded (n=4). Additionally, based on the study’s objectives, we further excluded participants with missing UHR data (n=398). Ultimately, 2,404 participants were included in the final analysis. The detailed study flow is illustrated in **Figure 1**.

### Ethics approval

According to Article 39 of the “Ethical Review Measures for Biomedical Research Involving Humans” issued by the National Health Commission of China, the informed consent exemption clause (37) stipulates: Research that utilizes human materials or data containing identifiable personal information may be exempt from the requirement to obtain signed informed consent, provided that the research does not involve personal privacy or commercial interests. In the current study, the design and implementation of the Jiangxi-ADHF II project were reviewed and approved by the Ethics Committee of Jiangxi Provincial People’s Hospital (Approval No. 2024-001), adhering strictly to the ethical principles outlined in the Declaration of Helsinki. Research data were recorded and analyzed in accordance with the Strengthening the Reporting of Observational Studies in Epidemiology guidelines after obtaining oral informed consent from participants or their legal representatives.

### Data collection

Baseline data collection and verification were completed within 24 hours of patient admission by a professionally trained two-member research team. The collected parameters included demographic characteristics (gender, age), New York Heart Association (NYHA) functional classification, comorbidities (hypertension, diabetes, stroke, and coronary heart disease [CHD]), echocardiographic left ventricular ejection fraction (LVEF), lifestyle history (smoking and drinking status), and laboratory test results. Specifically, comorbidities were confirmed through a comprehensive assessment that integrated patient self-reported medical history, current medication regimens, and past diagnostic records.

Laboratory tests were uniformly conducted within 24 hours of admission at the Clinical Laboratory Center of Jiangxi Provincial People’s Hospital. The tested parameters included NT-proBNP, complete blood count (white blood cell count: WBC; red blood cell count: RBC; platelet count), liver function markers (alanine aminotransferase, and aspartate aminotransferase: AST), UA, lipid profiles (total cholesterol, triglycerides, low-density lipoprotein cholesterol, and HDL-C), and fasting plasma glucose (FPG). Fasting blood samples were required for FPG, liver enzyme, and lipid profile tests, and specimens were collected either at admission or early the following morning.

### Calculation formula


UHR (%) = [UA (mg/dL)/HDL-C (mg/dL)] × 100

(27).

### Research outcomes

This study used 30-day all-cause mortality as the primary endpoint and cardiovascular mortality as the secondary endpoint. In the current study, cardiovascular deaths primarily included HF-related deaths, arrhythmia/sudden cardiac deaths, and stroke-related deaths; non-cardiovascular deaths primarily included septic shock/sepsis-related deaths, respiratory disease-related deaths, and other deaths attributed to clearly diagnosed non-cardiovascular causes. Follow-up procedures began on the day of patient admission, with survival status verified by uniformly trained researchers through multiple methods (including telephone follow-up, text message confirmation, and outpatient/inpatient follow-up visits).

### Handling of missing data

The covariates included in this study exhibited a low rate of missingness (0–4.70%, **Supplementary Table 1**). A missingness pattern plot (**Supplementary Figure 1**) confirmed that the data met the criteria for missing at random. Due to these characteristics and the low overall missingness rate, the final analysis was conducted using the original dataset without imputation.

### Statistical analysis

Data analysis was performed using R (version 4.2.1) and Empower(R) software (version 2.0). Baseline characteristics were described with appropriate statistical methods based on data types: categorical variables were presented as frequencies (percentages), normally distributed continuous variables as means (standard deviations), and non-normally distributed variables as medians (interquartile ranges). Group comparisons were conducted using one-way ANOVA, Kruskal-Wallis test, or chi-square test according to data characteristics, with a two-sided significance level set at α=0.05.

Kaplan-Meier analysis was used to plot survival curves for subjects stratified by UHR quartiles, and the log-rank test was applied to evaluate statistical differences. Multivariable Cox regression models were constructed to assess the association between UHR and prognosis in ADHF patients, and three levels of models were developed through stepwise adjustment of covariates: Model I (Basic Model): Adjusted for demographic factors (gender, age). Model II (Extended Model): Added lifestyle factors (smoking status, drinking status) and clinical characteristics (hypertension, diabetes, stroke, CHD) along with LVEF, building on Model I. Model III (Final Model): Further incorporated laboratory parameters (WBC, RBC, platelet count, AST, triglycerides, low-density lipoprotein cholesterol, FPG, and NT-proBNP) based on Model II. All models underwent variance inflation factor analysis to verify multicollinearity among covariates (**Supplementary Table 2**). In addition, the proportional hazards assumption of the model was tested using Schoenfeld residuals, with no violations detected.

Restricted cubic spline (RCS) analysis was employed (with 4 knots at the 5th, 35th, 65th, and 95th percentiles) to examine dose-response relationships between UHR and all-cause/cardiovascular mortality. Stratified analysis was further conducted to explore subgroup-specific associations of UHR with all-cause/cardiovascular mortality among ADHF patients. Likelihood ratio tests were used to evaluate linear/non-linear associations and test for differences across strata. Regarding the selection of knots in the RCS analysis, we referred to the recommendations in Professor Harrell’s book Regression Modeling Strategies (38): With 4 knots, the model achieves a good fit by balancing curve smoothness and avoiding reduced accuracy caused by overfitting. For larger sample sizes, 5 knots are preferable, while 3 knots are recommended for small samples (n < 30).

### Sensitivity analysis

Four sensitivity analyses were conducted across four dimensions to test the robustness of results:

1. Considering the potential impact of frailty on adverse outcomes (39), we defined a frailty subgroup as individuals with ≥ 3 comorbidities in this study; we excluded this subgroup and reanalyzed the data.
2. To mitigate potential reverse causality, we excluded participants who experienced death events within 48 hours of admission.
3. A quadratic term for age was incorporated into the final model to account for potential non-linear associations between age and adverse outcomes (40).
4. To reduce potential bias from missing data, we estimated missing values via multiple imputation and repeated the primary analysis.
5. To validate the generalizability of our findings, we conducted external validation using data from the 1998–2018 U.S. (United States) National Health and Nutrition Examination Survey; specifically, we examined the association between UHR and all-cause and cardiovascular mortality in congestive HF patients within this independent sample.
6. To address the potential confounding effects of medications, we further included urate-lowering therapy, statin therapy, and diuretic therapy as covariates and adjusted for these treatment factors in the multivariate model (Sensitivity-6). Additionally, we excluded patients undergoing urate-lowering therapy or statin therapy and adjusted for diuretic therapy in the multivariate analysis (Sensitivity-7).

## Results

### Baseline characteristics

According to the study population screening process, we excluded 160 patients with malignant tumors, 231 patients diagnosed with uremia or those with chronic kidney disease undergoing hemodialysis treatment, 42 patients with liver cirrhosis, 121 patients with pacemakers, 102 patients who recently underwent percutaneous coronary intervention treatment, 22 minor patients, 4 pregnant patients, and 398 patients with missing UHR data.

A total of 2,404 ADHF patients were included in this study, comprising 58.7% males (n=1,412) and 41.3% females (n=992). As shown in **Table 1**, comparisons across UHR quartiles revealed that patients in the highest UHR quartile, compared to the lowest quartile, exhibited the following characteristics: (1) Demographic characteristics: A higher proportion of males and younger age. (2) Laboratory parameters: Levels of LVEF, total cholesterol, and HDL-C were lower, while WBC, RBC, alanine aminotransferase, AST, UA, and NT-proBNP levels were significantly elevated. (3) Clinical characteristics: A higher proportion of patients presented with NYHA Class IV. Notably, comorbidity distribution showed no statistical difference across groups (*P*>0.05).

**Table 1:** Summary of baseline characteristics of the study population according to UHR quartiles group.

Variable	UHR quartiles				*P*-value
	Q1 10.60 (1.69-13.52)	Q2 16.27 (13.53-19.10)	Q3 22.33 (19.10-27.00)	Q4 35.00(27.00-169.27)	
No. of subjects	601	601	601	601	
Age (years)	72.00 (64.00-81.00)	72.00 (63.00-80.00)	69.00 (60.00-78.00)	69.00 (57.00-79.00)	<0.001
Gender (n,%)					<0.001
Male	253 (42.10%)	359 (59.73%)	391 (65.06%)	409 (68.05%)	
Female	348 (57.90%)	242 (40.27%)	210 (34.94%)	192 (31.95%)	
Hypertension (n,%)	263 (43.76%)	277 (46.09%)	283 (47.09%)	259 (43.09%)	0.457
Diabetes (n,%)	139 (23.13%)	155 (25.79%)	156 (25.96%)	179 (29.78%)	0.072
Stroke (n,%)	120 (19.97%)	95 (15.81%)	105 (17.47%)	91 (15.14%)	0.118
CHD (n,%)	183 (30.45%)	202 (33.61%)	211 (35.11%)	199 (33.11%)	0.381
NYHA classification (n,%)					<0.001
III	458 (76.21%)	437 (72.71%)	401 (66.72%)	335 (55.74%)	
IV	143 (23.79%)	164 (27.29%)	200 (33.28%)	266 (44.26%)	
Drinking status (n,%)	47 (7.82%)	59 (9.82%)	64 (10.65%)	70 (11.65%)	0.151
Smoking status (n,%)	87 (14.48%)	112 (18.64%)	108 (17.97%)	111 (18.47%)	0.185
LVEF (%)	50.00 (40.00-57.00)	48.00 (38.00-57.00)	45.00 (35.00-56.00)	42.00 (33.00-53.00)	<0.001
WBC (×10^9^/L)	5.80 (4.65-7.64)	5.80 (4.80-7.48)	6.40 (5.10-7.90)	6.65 (5.20-8.98)	<0.001
RBC (×10^12^/L)	3.98 (0.64)	4.03 (0.74)	4.11 (0.77)	4.14 (0.90)	0.001
PLT (×10^9^/L)	169.00 (128.00-214.00)	162.00 (126.00-204.00)	168.00 (128.00-217.00)	158.00 (118.00-212.00)	0.292
ALT (U/L)	21.00 (13.00-34.00)	18.00 (12.00-31.00)	23.00 (14.00-41.00)	25.00 (15.00-54.00)	<0.001
AST (U/L)	25.00 (19.00-34.00)	24.00 (18.00-34.00)	27.00 (20.00-41.00)	30.00 (21.00-51.00)	<0.001
UA (umol/L)	292.24 (82.40)	406.13 (81.01)	481.52 (100.91)	624.81 (154.41)	<0.001
TG (mmol/L)	1.02 (0.80-1.37)	1.09 (0.83-1.42)	1.20 (0.91-1.62)	1.24 (0.95-1.75)	<0.001
TC (mmol/L)	4.11 (3.50-4.74)	3.83 (3.22-4.59)	3.62 (3.10-4.28)	3.28 (2.68-4.01)	<0.001
HDL-C (mmol/L)	1.27 (0.29)	1.09 (0.22)	0.93 (0.19)	0.72 (0.19)	<0.001
LDL-C (mmol/L)	2.29 (1.82-2.79)	2.23 (1.74-2.86)	2.24 (1.77-2.84)	2.10 (1.60-2.72)	<0.001
FPG (mmol/L)	5.40 (4.70-6.15)	5.30 (4.60-6.10)	5.30 (4.70-6.20)	5.40 (4.70-6.40)	0.261
NT-proBNP (pmol/L)	2742.00 (1294.00-4706.00)	3295.00 (1733.00-5342.00)	3956.00 (1937.00-6519.00)	4934.00 (2831.00-8090.00)	<0.001
30-day all-cause mortality (n,%)	23 (3.83%)	25 (4.16%)	41 (6.82%)	67 (11.15%)	<0.001
30-day cardiovascular mortality (n,%)	14 (2.33%)	21 (3.49%)	32 (5.32%)	53 (8.82%)	<0.001

UHR, uric acid to high-density lipoprotein cholesterol ratio; CHD, coronary heart disease; NYHA, New York Heart Association; LVEF: left ventricular ejection fraction; TG, triglyceride; TC, total cholesterol; HDL-C, high-density lipoprotein cholesterol; LDL-C, low-density lipid cholesterol; WBC, white blood cell count; RBC, red blood cell count; PLT, platelet count; ALT, alanine aminotransferase; AST, aspartate aminotransferase; NT-proBNP, N-Terminal Pro-Brain Natriuretic Peptide; UA, uric acid; FPG, fasting plasma glucose; UHR, uric acid to high-density lipoprotein cholesterol ratio.

### Follow-up results

**Figure 2:**
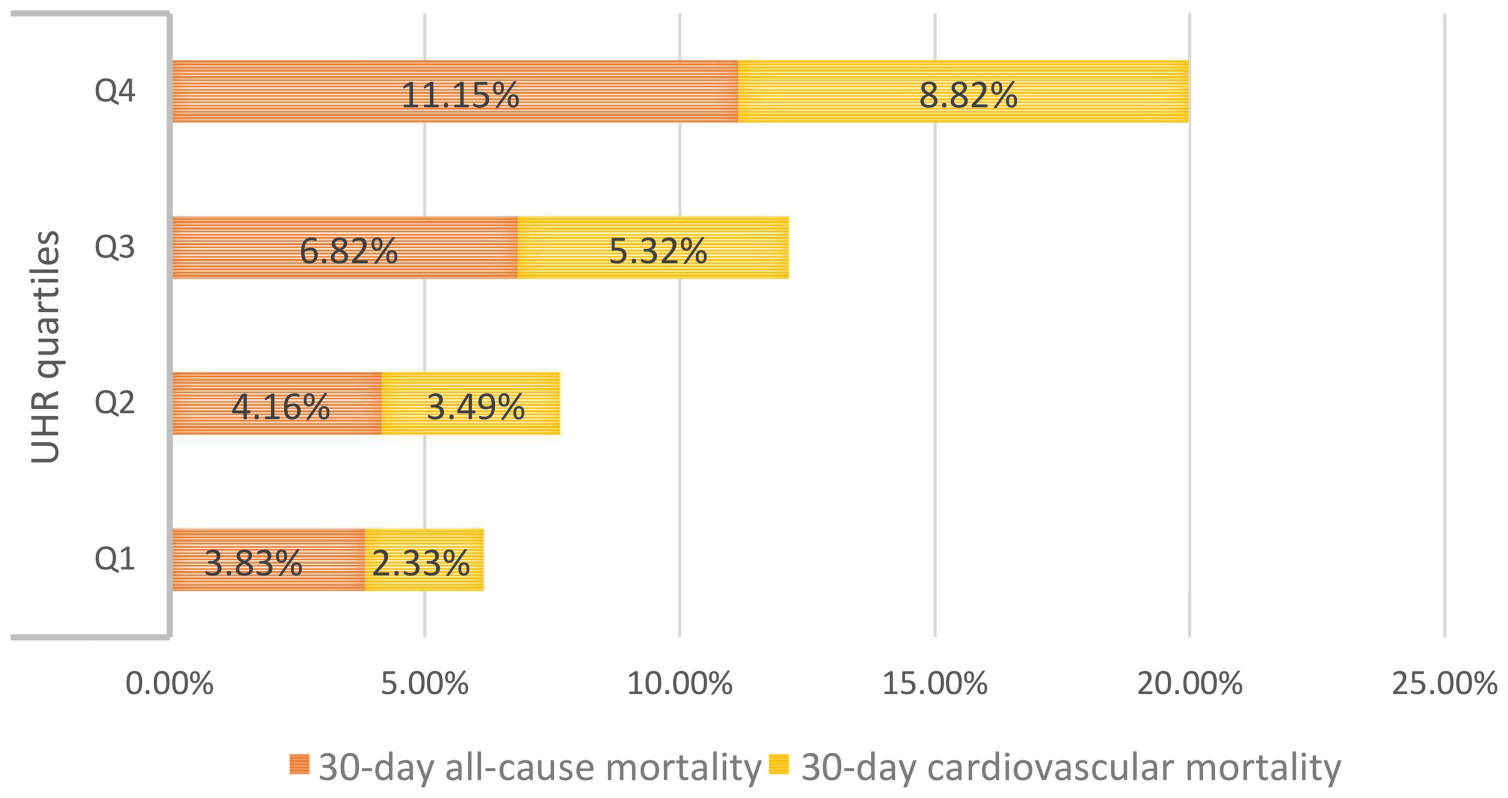
The stacked bar chart shows 30-day all-cause/cardiovascular mortality in ADHF patients according to UHR quartile groups. UHR: uric acid to high-density lipoprotein cholesterol ratio; ADHF: acute decompensated heart failure.

During the 30-day observation period, mortality events occurred in 156 participants (6.49%), with 120 cases (4.99%) attributed to cardiovascular causes. Based on UHR quartile grouping, we generated stacked bar charts for 30-day all-cause and cardiovascular mortality rates (**Figure 2**), and the results demonstrated a progressive increase in both all-cause and cardiovascular mortality within 30 days among ADHF patients as UHR quartiles rose.

**Figure 3:**
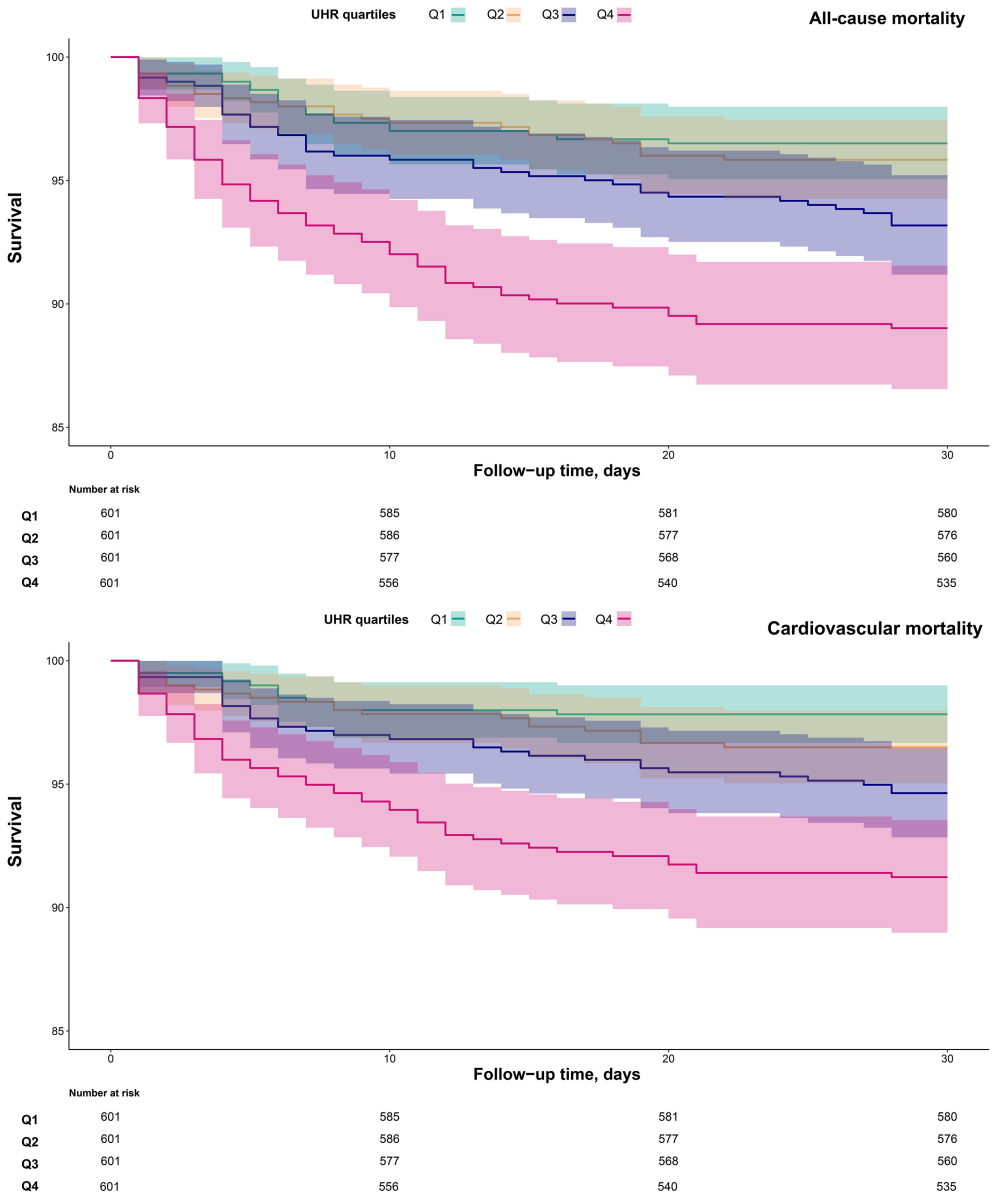
30-day survival curves of ADHF patients stratified by UHR quartiles. UHR, uric acid to high-density lipoprotein cholesterol ratio; ADHF, acute decompensated heart failure.

Additionally, 30-day survival curves stratified by UHR groups were plotted using the Kaplan-Meier method (**Figure 3**), revealing significantly higher mortality in the Q4 group compared to the other three groups (log-rank *p* < 0.0001).

### Association analysis

We incorporated UHR as both continuous and categorical variables into three progressively adjusted Cox regression models to investigate its association with 30-day all-cause and cardiovascular mortality in ADHF patients (**Table 2**). Whether treated as continuous or categorical variables, a positive association between UHR and both 30-day all-cause and cardiovascular mortality was observed across all adjusted models. In the fully adjusted model (Model III), each unit increase in UHR was associated with a 30% higher risk of all-cause mortality (Hazard ratio [HR]: 1.30, 1.17–1.45) and a 30% higher risk of cardiovascular mortality (HR: 1.30, 1.16–1.46) among ADHF patients. When UHR was analyzed as a categorical variable, the results showed that compared with the lowest quartile (Q1), individuals in the highest quartile (Q4) had significantly elevated risks of both all-cause and cardiovascular mortality. Specifically, in the final model, ADHF patients in Q4 experienced an 88% increased risk of all-cause mortality (HR: 1.88, 1.08–3.26) and a 113% increased risk of cardiovascular mortality (HR: 2.13, 1.06–4.28) within 30 days, compared with those in Q1.

**Table 2:** Multivariable Cox regression analysis of the association between UHR and 30-day all-cause and cardiovascular mortality in patients with ADHF.

Independent variable	HR (95%CI)			
	Non-adjusted	Model I	Model II	Model III
All-cause mortality				
UHR (Per SD increase)	1.48 (1.36, 1.60)	1.50 (1.39, 1.62)	1.37 (1.25, 1.51)	1.30 (1.17, 1.45)
UHR (quartiles)				
Q1	1.0	1.0	1.0	1.0
Q2	1.19 (0.67, 2.13)	1.18 (0.66, 2.12)	1.21 (0.66, 2.22)	1.25 (0.68, 2.31)
Q3	1.97 (1.17, 3.34)	2.13 (1.25, 3.63)	1.93 (1.11, 3.35)	1.68 (0.95, 2.96)
Q4	3.27 (2.00, 5.35)	3.61 (2.20, 5.94)	2.44 (1.43, 4.16)	1.88 (1.08, 3.26)
Cardiovascular mortality				
UHR (Per SD increase)	1.50 (1.38, 1.64)	1.51 (1.39, 1.65)	1.39 (1.26, 1.53)	1.30 (1.16, 1.46)
UHR (quartiles)				
Q1	1.0	1.0	1.0	1.0
Q2	1.62 (0.81, 3.23)	1.58 (0.79, 3.17)	1.69 (0.81, 3.54)	1.61 (0.76, 3.42)
Q3	2.49 (1.31, 4.74)	2.55 (1.33, 4.89)	2.44 (1.22, 4.89)	2.13 (1.05, 4.30)
Q4	4.17 (2.27, 7.66)	4.33 (2.34, 8.02)	2.97 (1.52, 5.84)	2.13 (1.06, 4.28)

HR, Hazard ratio; CI, confidence interval; ADHF, acute decompensated heart failure; SD, standard deviation; UHR, uric acid to high-density lipoprotein cholesterol ratio.

Model I adjusted for gender, age.

Model II adjusted for gender, age, drinking status, smoking status, hypertension, diabetes, stroke, CHD and LVEF.

Model III adjusted for gender, age, drinking status, smoking status, hypertension, diabetes, stroke, CHD, LVEF, WBC, RBC, PLT, AST, TG, LDL-C, FPG, NT-proBNP.

### Dose-response relationship

**Figure 4:**
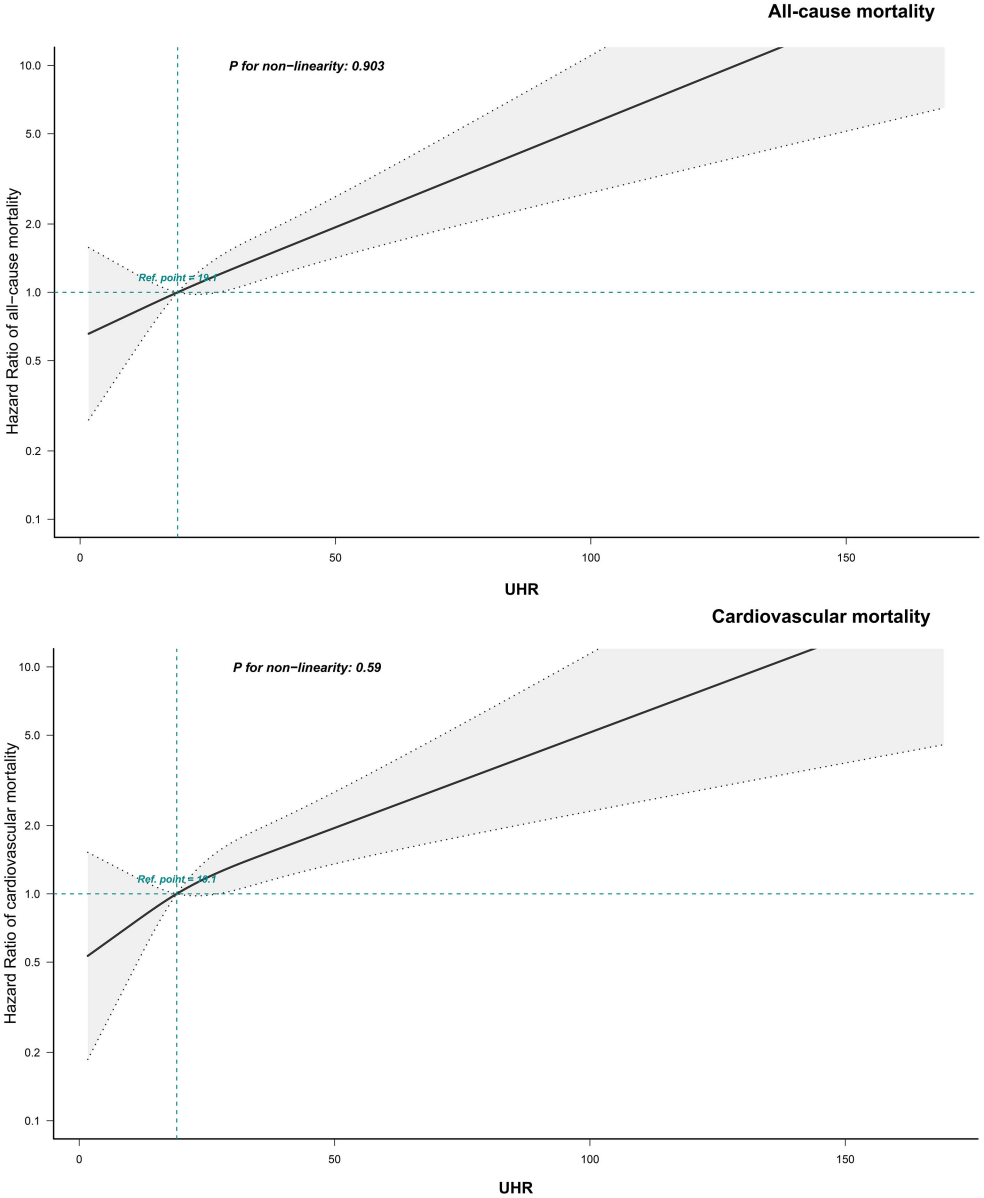
Fitting the dose-response relationship between UHR and 30-day all-cause/cardiovascular mortality in ADHF patients with 4 knots restricted cubic spline. UHR, uric acid to high-density lipoprotein cholesterol ratio; ADHF, acute decompensated heart failure. Adjusted for gender, age, drinking status, smoking status, hypertension, diabetes, stroke, CHD, LVEF, WBC, RBC, PLT, AST, TG, LDL-C, FPG, NT-proBNP.

To further investigate the association between UHR and mortality outcomes, adjusted RCS curves were used to visualize the dose-response relationships of UHR with all-cause and cardiovascular mortality (**Figure 4**). The analysis revealed a linear positive association between UHR and both all-cause mortality (*P* for non-linearity = 0.903) and cardiovascular mortality (*P* for non-linearity = 0.590) in ADHF patients.

### Subgroup analysis

**Figure 5:**
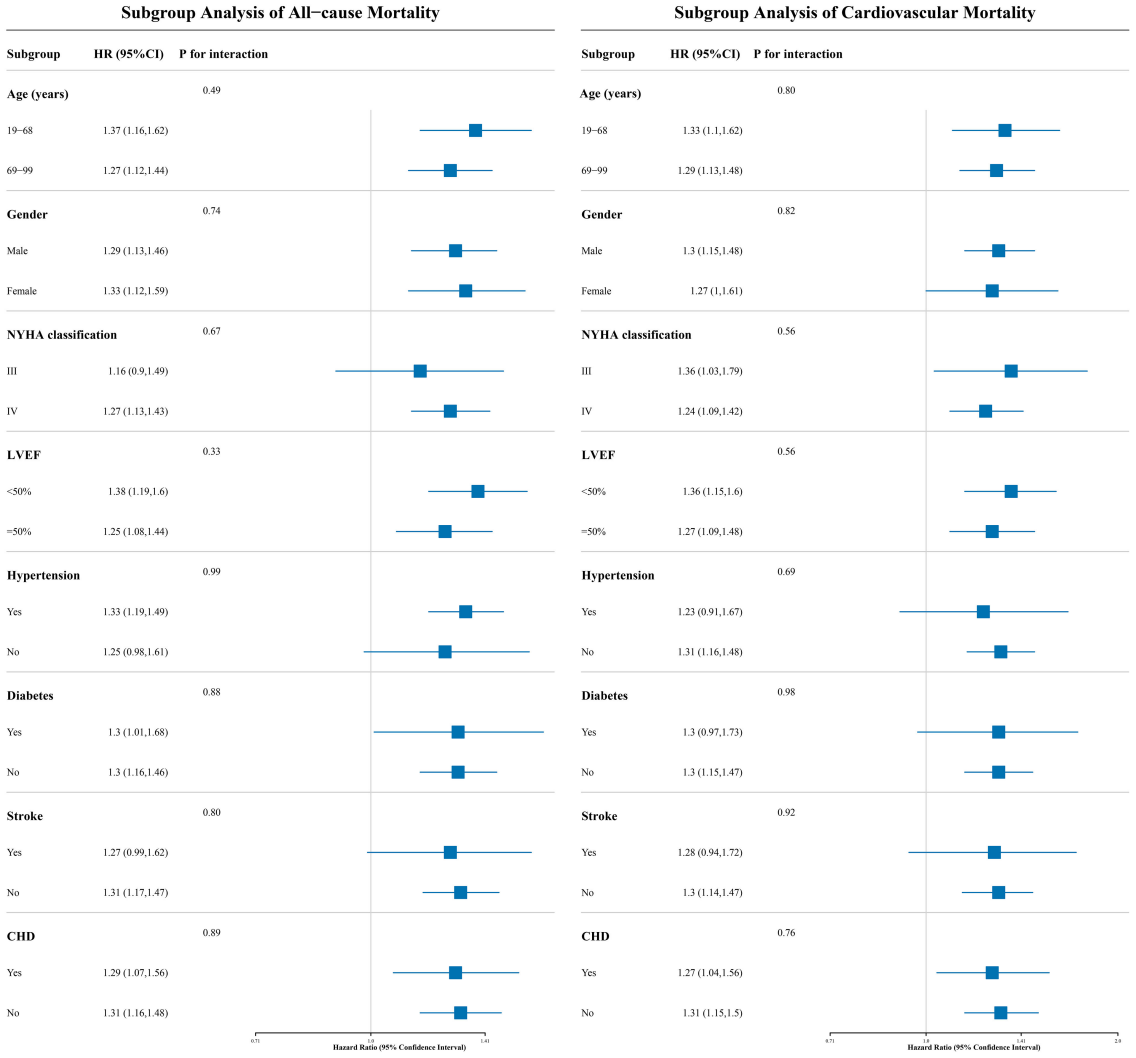
The forest plot shows the association between UHR and 30-day all-cause and cardiovascular mortality in ADHF patients across different subgroups. UHR, uric acid to high-density lipoprotein cholesterol ratio; ADHF, acute decompensated heart failure. Adjusted for gender, age, drinking status, smoking status, hypertension, diabetes, stroke, CHD, LVEF, WBC, RBC, PLT, AST, TG, LDL-C, FPG, NT-proBNP.

This study employed a stratified analysis approach to stratify patients into subgroups based on demographic characteristics (age, gender), clinical indicators (NYHA classification, LVEF), and comorbidities (**Table 3**). The likelihood ratio test was used to evaluate interaction effects between UHR and each stratifying factor, and the results showed no significant interactions across all subgroups (all *P* for interaction > 0.05; **Figure 5** for details). This finding suggests that the association between UHR and adverse outcomes (all-cause and cardiovascular mortality) in ADHF patients is consistent across subgroups and not influenced by the aforementioned clinical characteristics.

**Table 3:** Stratified analysis showed the relationship between UHR and 30-day mortality in patients with ADHF in different age, gender, NYHA classification, LVEF and whether combined with hypertension/diabetes/stroke/CHD.

Subgroup	HR Per SD increase (95%CI)	
	All-cause mortality	Cardiovascular mortality
Age (years)		
19-68	1.37 (1.16, 1.62)	1.33 (1.10, 1.62)
69-99	1.27 (1.12, 1.44)	1.29 (1.13, 1.48)
*P* for interaction	0.49	0.80
Gender		
Male	1.29 (1.13, 1.46)	1.30 (1.15, 1.48)
Female	1.33 (1.12, 1.59)	1.27 (1.00, 1.61)
*P* for interaction	0.74	0.82
NYHA classification		
III	1.16 (0.90, 1.49)	1.36 (1.03, 1.79)
IV	1.27 (1.13, 1.43)	1.24 (1.09, 1.42)
*P* for interaction	0.67	0.56
LVEF		
< 50%	1.38 (1.19, 1.60)	1.36 (1.15, 1.60)
≥ 50%	1.25 (1.08, 1.44)	1.27 (1.09, 1.48)
*P* for interaction	0.33	0.56
Hypertension		
Yes	1.33 (1.19, 1.49)	1.23 (0.91, 1.67)
No	1.25 (0.98, 1.61)	1.31 (1.16, 1.48)
*P* for interaction	0.99	0.69
Diabetes		
Yes	1.30 (1.01, 1.68)	1.30 (0.97, 1.73)
No	1.30 (1.16, 1.46)	1.30 (1.15, 1.47)
*P* for interaction	0.88	0.98
Stroke		
Yes	1.27 (0.99, 1.62)	1.28 (0.94, 1.72)
No	1.31 (1.17, 1.47)	1.30 (1.14, 1.47)
*P* for interaction	0.80	0.92
CHD		
Yes	1.29 (1.07, 1.56)	1.27 (1.04, 1.56)
No	1.31 (1.16, 1.48)	1.31 (1.15, 1.50)
*P* for interaction	0.89	0.76

UHR, uric acid to high-density lipoprotein cholesterol ratio; ADHF, acute decompensated heart failure; CHD, coronary heart disease; NYHA, New York Heart Association; SD, standard deviation; HR, Hazard ratio; CI, confidence interval.

Models adjusted for the same covariates as in model III (**Table 2**), except for the stratification variable.

### Sensitivity analysis

First, after excluding frail subgroup patients and re-analyzing the data, we observed results consistent with the primary analysis. Second, re-analysis after excluding participants who died within 48 hours of admission yielded findings aligned with the primary analysis. Third, further adjustment for the quadratic term of age did not significantly alter the results. Fourth, validation analysis in the imputed complete dataset confirmed the robustness of the findings (**Table 4**). Fifth, regarding UHR distribution, our study cohort and the U.S. HF cohort exhibited comparable distribution patterns (**Supplementary Figure 2**). In terms of association analysis, results from the U.S. HF cohort indicated a positive association between UHR and both all-cause mortality and cardiovascular mortality. These findings were consistent with the results of the present study. Sixth, after accounting for the potential influences of urate-lowering therapy, statin therapy, and diuretic therapy, the new findings remained consistent with the primary results (Sensitivity-6 and Sensitivity-7).

**Table 4:** Sensitivity analysis.

Independent variable	HR (95% CI)						
	Sensitivity-1	Sensitivity-2	Sensitivity-3	Sensitivity-4	Sensitivity-5	Sensitivity-6	Sensitivity-7
All-cause mortality							
UHR (Per SD increase)	1.28 (1.15, 1.43)	1.25 (1.10, 1.43)	1.30 (1.17, 1.44)	1.33 (1.21, 1.46)	1.21 (1.12, 1.30)	1.27 (1.14, 1.41)	1.22 (1.05, 1.41)
UHR (quartiles)							
Q1	Ref	Ref	Ref	Ref	Ref	Ref	Ref
Q2	1.17 (0.58, 2.36)	1.13 (0.57, 2.26)	1.27 (0.69, 2.35)	1.22 (0.68, 2.19)	0.96 (0.78, 1.18)	1.32 (0.71, 2.44)	0.88 (0.36, 2.18)
Q3	1.48 (0.78, 2.81)	1.87 (1.01, 3.48)	1.70 (0.96, 3.00)	1.70 (0.99, 2.92)	1.02 (0.83, 1.25)	1.80 (1.02, 3.19)	1.08 (0.46, 2.51)
Q4	1.79 (0.96, 3.33)	1.97 (1.07, 3.62)	1.93 (1.11, 3.35)	2.12 (1.26, 3.56)	1.28 (1.04, 1.57)	2.05 (1.17, 3.58)	1.64 (0.75, 3.58)
*P*-trend	0.04	0.01	0.01	<0.01	<0.01	<0.01	0.15
Cardiovascular mortality							
UHR (Per SD increase)	1.29 (1.14, 1.45)	1.23 (1.06, 1.44)	1.30 (1.16, 1.46)	1.32 (1.19, 1.47)	1.25 (1.11, 1.40)	1.28 (1.14, 1.45)	1.27 (1.06, 1.54)
UHR (quartiles)							
Q1	Ref	Ref	Ref	Ref	Ref	Ref	Ref
Q2	1.54 (0.67, 3.54)	1.51 (0.65, 3.51)	1.64 (0.77, 3.48)	1.51 (0.75, 3.04)	1.02 (0.73, 1.42)	1.69 (0.80, 3.58)	0.98 (0.32, 2.99)
Q3	1.96 (0.90, 4.29)	2.44 (1.12, 5.29)	2.21 (1.09, 4.47)	1.95 (1.01, 3.77)	1.08 (0.77, 1.51)	2.26 (1.12, 4.58)	1.51 (0.54, 4.20)
Q4	2.23 (1.04, 4.78)	2.34 (1.08, 5.08)	2.21 (1.10, 4.43)	2.32 (1.22, 4.41)	1.39 (1.01, 1.94)	2.37 (1.17, 4.78)	1.64 (0.62, 4.37)
*P*-trend	0.03	0.02	0.02	<0.01	<0.01	<0.01	0.22

UHR, uric acid to high-density lipoprotein cholesterol ratio; ADHF, acute decompensated heart failure; SD, standard deviation; HR, Hazard ratio; CI, confidence interval.

Adjusted for gender, age, drinking status, smoking status, hypertension, diabetes, stroke, CHD, LVEF, WBC, RBC, PLT, AST, TG, LDL-C, FPG, NT-proBNP.

Note 1: Hypertension, diabetes, stroke and CHD were not adjusted in Sensitivity-1.

Note 2: gender, age, drinking status, smoking status, hypertension, diabetes, stroke, CHD were adjusted in Sensitivity-5.

Note 3: gender, age, drinking status, smoking status, hypertension, diabetes, stroke, CHD, LVEF, WBC, RBC, PLT, AST, TG, LDL-C, FPG, NT-proBNP, urate-lowering therapy, statin therapy, and diuretic therapy were adjusted in Sensitivity-6.

Note 4: gender, age, drinking status, smoking status, hypertension, diabetes, stroke, CHD, LVEF, WBC, RBC, PLT, AST, TG, LDL-C, FPG, NT-proBNP, and diuretic therapy were adjusted in Sensitivity-7.

## Discussion

In this retrospective cohort study of ADHF patients in Jiangxi, China, we demonstrated for the first time a linear positive association between UHR and short-term (30-day) all-cause and cardiovascular mortality. The robustness of these findings was validated across four dimensions: population heterogeneity, causal temporality, model adjustment, and data integrity.

UA is generated as the final product of purine metabolism and exhibits a dual role in oxidative stress, acting as both an antioxidant and a pro-oxidant (41–43). On one hand, UA functions as a potent antioxidant by donating hydrogen atoms to free radicals, thereby stabilizing them and preventing oxidative damage. Additionally, UA exerts cardiovascular protective effects by inhibiting superoxide radical-mediated degradation of nitric oxide, which promotes vasodilation (44–47). On the other hand, elevated UA levels can induce inflammation and activate the renin-angiotensin-aldosterone system, ultimately contributing to vascular endothelial dysfunction, oxidative stress, and various inflammatory responses (48–51). In recent years, due to significant dietary shifts and rising prevalence of unhealthy lifestyles, the incidence of hyperuricemia has gradually risen. According to statistics, approximately 170 million people in China suffer from hyperuricemia, posing a significant impact on cardiovascular health (52–54). HDL-C is a critical component of lipid metabolism and exhibits a complex relationship with cardiovascular events. Traditionally, the classical theory posited a significant inverse association between HDL-C levels and cardiovascular risk (55, 56). However, with advances in research, recent studies have raised doubts about the protective role of elevated HDL-C in cardiovascular outcomes (57, 58). For instance, in a multicenter prospective cohort study based on the United Kingdom Biobank and the Emory Cardiovascular Biorepository, Liu et al. observed a significant increase in all-cause and cardiovascular mortality among patients when HDL-C levels were ≥ 80 mg/dL (59). This phenomenon may be attributed to conformational and functional alterations of HDL-C at extremely high levels, leading to a loss of its anti-inflammatory and antioxidant properties under inflammatory or oxidative stress conditions (60). Additionally, a meta-analysis of 37 prospective cohort studies revealed a J-shaped association between HDL-C levels and all-cause mortality, with the lowest risk observed at HDL-C concentrations between 54–58 mg/dL (61). Notably, recent research has begun to focus on potential interactions between UA and HDL-C: studies indicate that the combined effect of elevated UA and HDL-C levels may increase mortality risk in cardiovascular populations (25). Therefore, further elucidating the synergistic relationship between UA and HDL-C could be essential for evaluating adverse prognostic outcomes.

As a composite indicator of UA and HDL-C, the UHR was first proposed by Kocak et al. as a metabolic assessment parameter. In their initial study, Kocak et al. demonstrated that UHR exhibits high accuracy in diagnosing metabolic syndrome (27). Subsequently, an increasing number of studies have identified UHR as a significant influencing factor in cardiovascular disease risk assessment, including hypertension, CHD, and HF (28–32). In terms of HF risk assessment, data from the National Health and Nutrition Examination Survey revealed that compared to the low UHR levels (Q1), high UHR levels (Q4) were associated with a 159% increased risk of chronic HF (32). In the current study, we further demonstrated a significant positive correlation between UHR and both all-cause and cardiovascular mortality in Chinese patients with ADHF, thereby expanding the research evidence on UHR’s role in HF prognosis. Collectively, UHR emerges as an important factor in both HF risk stratification and prognostic evaluation, suggesting that incorporating UHR into cardiovascular disease screening protocols may offer significant clinical benefits. It should be noted that UHR, in addition to its utility in assessing mortality risk among HF patients, also plays an important role in evaluating death risk across various chronic disease populations and middle-aged/elderly cohorts (62–66). Specifically, studies have demonstrated the following: Among the United States (US) adult populations, high UHR levels (Q4) were associated with a 16-36% increased risk of all-cause mortality and a 20-37% elevated risk of cardiovascular mortality during long-term follow-up (63, 64). In patients with diabetes or prediabetes, high UHR conferred a 24% higher all-cause mortality risk and a 34-56% increased cardiovascular mortality risk (65, 66). Among peritoneal dialysis patients, high UHR was linked to a 35% greater risk of all-cause death and a 46% higher cardiovascular mortality risk (65, 66). In the current study, we further demonstrated that among ADHF patients, high UHR levels were associated with an 88% increased risk of all-cause mortality and a 113% elevated risk of cardiovascular mortality. Compared to previous studies investigating UHR’s association with mortality across diverse populations (62–66), UHR appears to offer additional prognostic information specifically for ADHF patients, while also expanding the evidence base for its application in acute disease prognosis. Additionally, in the current study, we further explored the impact of multiple subgroup factors on the association between UHR and mortality outcomes. The results showed that no significant interaction effects were detected across all subgroups. This finding further suggests that UHR may have high generalizability for assessing adverse prognosis in ADHF patients in Jiangxi. Certainly, after subgroup stratification, the sample size for each stratum decreases relatively; this may also lead to potential false-negative results in the subgroup analyses in the current study, as detecting interaction effects typically requires a much larger sample size than detecting main effects. Interaction effects are assessed by comparing differences between effect sizes; such differences exhibit greater variability and require more data to stabilize the estimation (67, 68). A widely cited rule of thumb states that detecting interaction effects requires a sample size at least four times larger than that needed to detect main effects of comparable magnitude.

In the current study, we further demonstrated through RCS analysis that UHR exhibits a linear association with both all-cause mortality (*P* for non-linearity = 0.903) and cardiovascular mortality (*P* for non-linearity = 0.590) among ADHF patients. Notably, similar linear findings were reported in recent studies (32, 63): In a cross-sectional study conducted by Yi et al., a linear association was observed between UHR and chronic HF (*P* for non-linearity = 0.564) among U.S. adults. Additionally, among middle-aged and elderly U.S. populations, UHR demonstrated a linear association with cardiovascular mortality (*P* for non-linearity = 0.408). However, inconsistent findings were also reported in other UHR-related studies: Lai et al. observed a J-shaped association between UHR and both all-cause and cardiovascular mortality among U.S. patients with diabetes or prediabetes (65), with calculated threshold values of 13.73 and 9.39, respectively. In contrast, Huang et al. demonstrated a U-shaped relationship between UHR and mortality outcomes in U.S. diabetic patients (66), where excessively high or low UHR levels were associated with increased mortality risk. The discrepancies between our current findings and UHR-related prognostic outcomes in U.S. populations may be attributed to racial differences and disease heterogeneity, and further studies across diverse populations are warranted to validate these associations.

Adverse outcomes during hospitalization for ADHF patients have consistently been one of the most critical clinical challenges that require resolution. Current research evidence indicates that the UHR serves as an independent predictor of mortality prognosis in ADHF patients. From a computational perspective, UHR is influenced by UA and HDL-C levels; when UHR increases, the balance between these two biomarkers becomes disrupted. This imbalance leads to the accumulation of numerous detrimental factors, which may cause vascular damage and atherosclerosis (28–31), ultimately resulting in HF progression and even death (32). From a mechanistic perspective, elevated UA and low HDL-C share a common pathological basis in promoting the development of HF, primarily including oxidative damage and inflammatory damage to endothelial cells (19, 69–71). High levels of UA activate nicotinamide adenine dinucleotide phosphate oxidase and induce mitochondrial dysfunction, leading to a surge in reactive oxygen species (ROS). These ROS subsequently activate the nuclear factor-kappa B and activator protein-1 pathways, promote renin release and angiotensin-converting enzyme synthesis, thereby forming a ROS-RAS positive feedback loop that exacerbates oxidative stress, inflammation, and endothelial dysfunction (69, 72). HDL is a lipoprotein with multiple protective functions (18, 19); its reduced levels can lead to impaired reverse cholesterol transport, attenuated anti-inflammatory and antioxidant capacities, and endothelial dysfunction, thereby exacerbating cardiac dysfunction (70, 71, 73). Notably, under the stress and inflammatory state of HF exacerbation, the function of HDL particles may shift from protective to pathological; this significantly reduces their antioxidant capacity and exacerbates oxidative stress-induced damage to endothelial cells and cardiomyocytes. In addition, during HF, particularly in the acute decompensated phases, impaired cardiac pumping function reduces effective blood ejection, leading to fluid retention and volume overload. This condition can initiate or worsen renal dysfunction, which in turn causes abnormal metabolism/dysfunction of UA and HDL-C, thereby diminishing their cardiovascular protective effects (73–76). Overall, elevated UA and low HDL-C are not isolated risk factors for ADHF progression; they may act synergistically through multiple mechanisms—such as amplifying inflammation and oxidative stress, exacerbating endothelial dysfunction, and promoting myocardial remodeling and fibrosis—to collectively contribute to the deterioration of cardiac function and adverse outcomes in patients with ADHF.

Notably, researchers in recent reports have evaluated the prognostic benefits of urate-lowering therapy or HDL-C-elevating interventions in HF patients (77, 78). A United Kingdom clinical practice study demonstrated that HF patients receiving urate-lowering treatment (including allopurinol, febuxostat, probenecid, lesinurad, pegloticase, and lesinurad/allopurinol combination) exhibited a significantly reduced risk of adverse clinical outcomes within 5 years (77). Furthermore, results from a multicenter randomized trial in Japan showed that urate-lowering therapy significantly improved 3-year adverse prognosis in HF patients; in contrast, febuxostat demonstrated greater efficacy than allopurinol in treating HF patients with comorbid hyperuricemia (78). Regarding HDL-C-elevating therapy for HF, experimental studies have demonstrated that recombinant HDL infusion improves cardiac diastolic function and attenuates myocardial fibrosis in mice (79); moreover, recombinant HDL infusion also showed significant electrophysiological improvements in HF treatment: electrocardiographic results indicated that recombinant HDL infusion shortened the duration of ventricular electrical systole, manifested as a reduction in heart rate-corrected QT interval (80). Overall, HDL-targeted therapy exerts direct positive inotropic effects on the myocardium, inhibits the development of cardiac hypertrophy, suppresses interstitial and perivascular myocardial fibrosis, increases myocardial capillary density, and thereby inhibits the occurrence and progression of HF (81). These findings collectively demonstrate the significant role of UHR in assessing cardiovascular disease risk and prognosis. Targeting UA reduction or HDL-C elevation may improve clinical outcomes in ADHF patients. However, whether the use of urate-lowering medications or interventions to raise HDL-C provides prognostic benefits in ADHF remains to be quantitatively evaluated in future clinical trials. Such studies should assess the comprehensive benefits of different drug combinations and dosages in HF patients.

The results of this study have important implications for the clinical management of ADHF. The UHR index, due to its testing simplicity (using existing laboratory assays) and cost-effectiveness, is particularly suitable for widespread adoption across healthcare institutions at all levels. Based on empirical evidence, we recommend integrating UHR into the ADHF risk stratification system as a supplementary tool to optimize clinical decision-making. The recommended approach is as follows: By automatically classifying patients’ UHR levels, high-risk ADHF patients at risk of disease progression could be identified early, thereby optimizing intervention decisions. Furthermore, UHR could be integrated into existing risk scoring models and embedded into artificial intelligence-assisted decision-making systems to dynamically predict the probability of adverse mortality outcomes in patients.

Our study has several notable strengths: (1) This is the first report of a linear positive correlation between UHR and both all-cause and cardiovascular mortality in Chinese ADHF patients. These novel findings provide new insights into early risk stratification for ADHF patients. Although we have further obtained similar results based on a U.S. HF cohort, validation in more ethnically diverse cohorts remains necessary. Additionally, exploring improved risk stratification strategies by integrating established risk scores (e.g., ADHFRE, MAGGIC) is also essential. (2) UHR is calculated using UA and HDL-C, both of which are routinely measured in standard health check-ups and easily accessible. The findings of this study therefore hold substantial practical value. Several limitations of this study need to be explained: (1) All study participants were recruited from Jiangxi Province, China, which may limit the generalizability of our findings due to the limited geographic and ethnic diversity of the sample. (2) Dynamic monitoring data of UHR were lacking in the current study, making it difficult to evaluate its temporal variation characteristics and predictive value as a short-term prognostic biomarker. (3) Although the analysis revealed a significant correlation between UHR levels and mortality risk in ADHF patients, the observational design of this study limits the findings to suggesting a potential association rather than establishing a definitive causal relationship. This limitation underscores the need for future mechanistic studies or randomized controlled trials to further validate these results. (4) Due to the retrospective design of this study, some critical disease-related indicators were not fully recorded (e.g., nutritional status, inflammatory markers like C-reactive protein/Interleukin 6). Although we implemented rigorous control of confounding factors through methods such as multivariable adjustment in statistical analyses, residual confounding from unmeasured variables may persist due to limitations of the study design, potentially influencing the study conclusions.

## Conclusion

In this retrospective cohort study, we first demonstrated that UHR could be utilized for the early assessment of short-term all-cause and cardiovascular mortality risk in ADHF patients, and that a high UHR was associated with an increased short-term all-cause and cardiovascular mortality risk in this population.

## Data Availability

The raw data supporting the conclusions of this article will be made available by the authors, without undue reservation.
