# Supplementary material for: Impact of serum uric acid to high-density lipoprotein cholesterol ratio on short-term outcomes in acute decompensated heart failure: a cohort study in Jiangxi Province, China
Source: Front Endocrinol (Lausanne). 2025 Oct 7;16:1667929. doi: 10.3389/fendo.2025.1667929 (PMC12537372; doi:10.3389/fendo.2025.1667929)
Supplement: Supplementary file 1 [file Table1.docx]

Supplementary Table 1. The missing number and rate of covariates.

| Variables | Non- Missing | Missing |
| --- | --- | --- |
| Gender | 2404 | 0 |
| Age | 2404 | 0 |
| Hypertension | 2404 | 0 |
| Diabetes | 2404 | 0 |
| Stroke | 2404 | 0 |
| CHD | 2404 | 0 |
| NYHA classification | 2404 | 0 |
| Drinking status | 2404 | 0 |
| Smoking status | 2404 | 0 |
| LVEF | 2291 | 113 |
| WBC | 2386 | 18 |
| RBC | 2386 | 18 |
| PLT | 2386 | 18 |
| ALT | 2390 | 14 |
| AST | 2392 | 12 |
| UA | 2404 | 0 |
| TG | 2404 | 0 |
| TC | 2404 | 0 |
| HDL-C | 2404 | 0 |
| LDL-C | 2404 | 0 |
| FPG | 2302 | 102 |
| NT-proBNP | 2404 | 0 |
| UHR | 2404 | 0 |

Abbreviations as in Table 1.

Supplementary Table 2: Collinearity diagnostics steps.

|  | VIF | |
| --- | --- | --- |
|  | Step 1 | Step 2 |
| UHR | 4.3 | 4.3 |
| Gender | 1.2 | 1.2 |
| Age | 1.4 | 1.4 |
| Hypertension | 1.1 | 1.1 |
| Diabetes | 1.4 | 1.4 |
| Stroke | 1.1 | 1.1 |
| CHD | 1.1 | 1.1 |
| NYHA classification | 1.1 | 1.1 |
| Drinking status | 1.5 | 1.5 |
| Smoking status | 1.6 | 1.6 |
| LVEF | 1.2 | 1.2 |
| WBC | 1.3 | 1.3 |
| RBC | 1.3 | 1.3 |
| PLT | 1.2 | 1.2 |
| ALT | 7.3 | NA |
| AST | 7.1 | 1.1 |
| UA | 2.7 | 2.7 |
| TG | 1.4 | 1.4 |
| TC | 1.8 | 1.7 |
| HDL-C | 2.6 | 2.6 |
| LDL-C | 1 | 1 |
| FPG | 1.4 | 1.4 |
| NT-proBNP | 1.2 | 1.2 |

VIF: variance inflation factor; VIF = 1/(1-R^2^). Abbreviations as in Table ​1.

Note: The variables with VIF>5 will be regarded as collinear variables.
