# Supplementary material for: Impact of serum uric acid to high-density lipoprotein cholesterol ratio on short-term outcomes in acute decompensated heart failure: a cohort study in Jiangxi Province, China
Source: Front Endocrinol (Lausanne). 2025 Oct 7;16:1667929. doi: 10.3389/fendo.2025.1667929 (PMC12537372; doi:10.3389/fendo.2025.1667929)
Supplement: Supplementary file 2 [file Table2.docx]

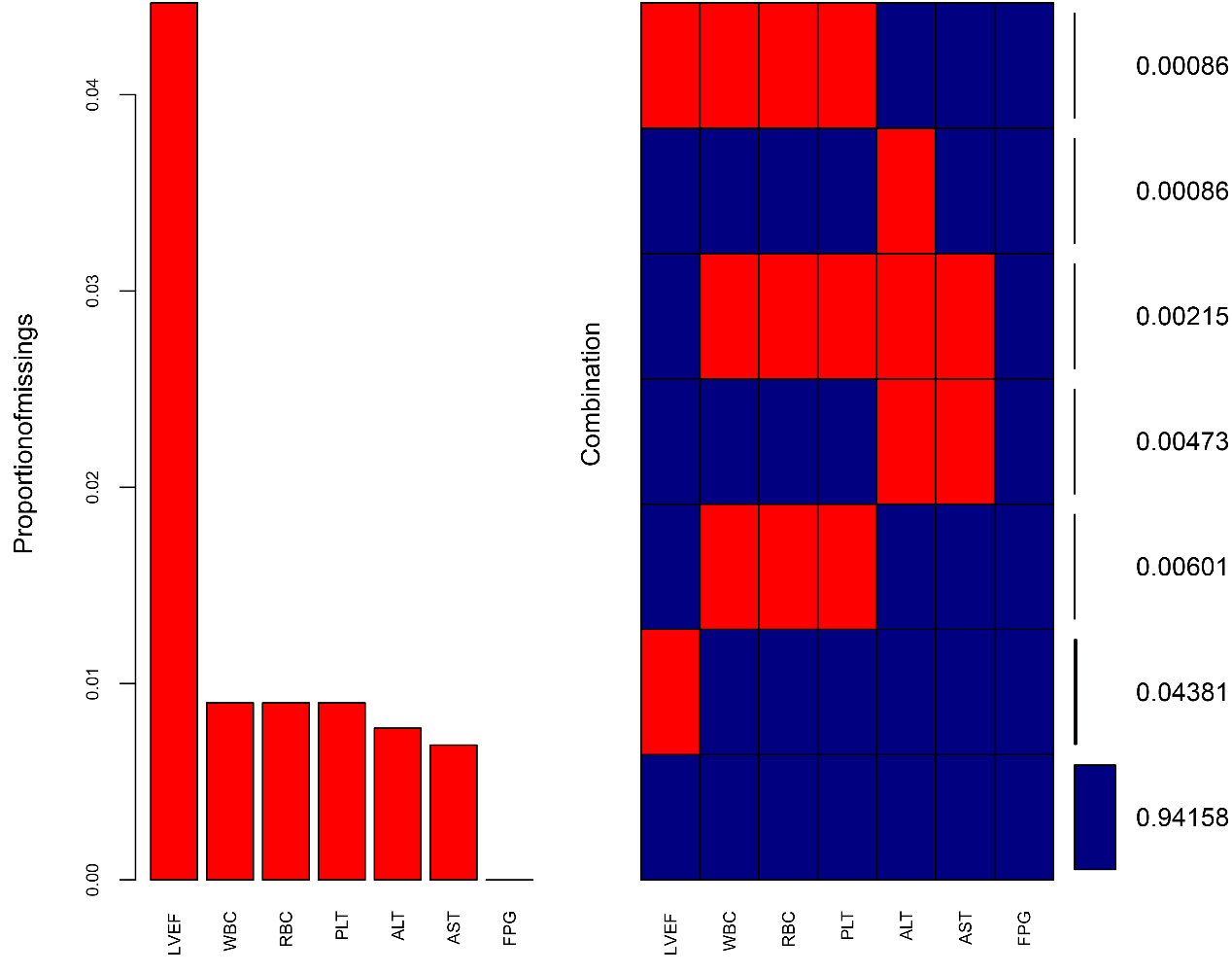


**Supplementary Figure 1:** Missing data cross-information diagram. LVEF: left ventricular ejection fraction; ALT: alanine aminotransferase; AST: aspartate aminotransferase; WBC: white blood cell count; RBC: red blood cell count; PLT: platelet count; FPG: fasting plasma glucose.


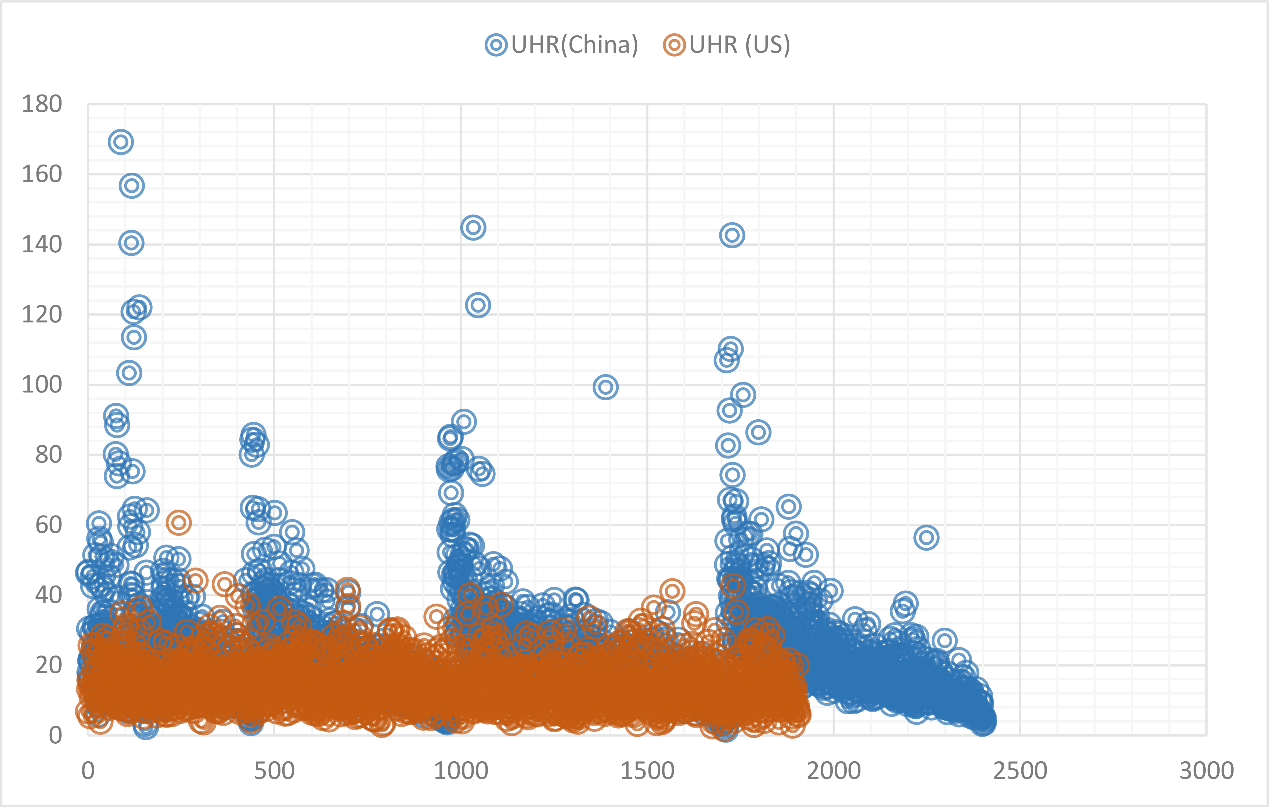


**Supplementary Figure 2:** Scatter Plot of UHR in the Jiangxi-ADHF cohort (China) and the NHANES Population (US).
